# Aberration of the modulatory functions of intronic microRNA hsa-miR-933 on its host gene *ATF2* results in type II diabetes mellitus and neurodegenerative disease development

**DOI:** 10.1186/s40246-020-00285-1

**Published:** 2020-09-29

**Authors:** Abul Bashar Mir Md. Khademul Islam, Eusra Mohammad, Md. Abdullah-Al-Kamran Khan

**Affiliations:** 1 Department of Genetic Engineering and Biotechnology, University of Dhaka, Dhaka, Bangladesh; 2 Current Affiliation: Department of Molecular Biology, Max Planck Institute for Biophysical Chemistry, Göttingen, Germany; 3 Current Affiliation: Department of Mathematics and Natural Sciences, BRAC University, Dhaka, Bangladesh

**Keywords:** MicroRNA, Intronic microRNA, hsa-miR-933, *ATF2*, diabetes mellitus, Neurodegenerative diseases

## Abstract

**Background:**

MicroRNAs are ~ 22-nucleotide-long biological modifiers that act as the post-transcriptional modulator of gene expression. Some of them are identified to be embedded within the introns of protein-coding genes, these miRNAs are called the intronic miRNAs. Previous findings state that these intronic miRNAs are co-expressed with their host genes. This co-expression is necessary to maintain the robustness of the biological system. Till to date, only a few experiments are performed discretely to elucidate the functional relationship between few co-expressed intronic miRNAs and their associated host genes.

**Results:**

In this study, we have interpreted the underlying modulatory mechanisms of intronic miRNA hsa-miR-933 on its target host gene *ATF2* and found that aberration can lead to several disease conditions. A protein-protein interaction network-based approach was adopted, and functional enrichment analysis was performed to elucidate the significantly over-represented biological functions and pathways of the common targets. Our approach delineated that hsa-miR-933 might control the hyperglycemic condition and hyperinsulinism by regulating ATF2 target genes *MAP4K4*, *PRKCE*, *PEA15*, *BDNF*, *PRKACB*, and *GNAS* which can otherwise lead to the development of type II diabetes mellitus. Moreover, we showed that hsa-miR-933 can regulate a target of ATF2, brain-derived neurotrophic factor (BDNF), to modulate the optimal expression of *ATF2* in neuron cells to render neuroprotection for the inhibition of neurodegenerative diseases.

**Conclusions:**

Our in silico model provides interesting resources for experimentations in a model organism or cell line for further validation. These findings may extend the common perception of gene expression analysis with new regulatory functionality.

## Background

MicroRNAs (miRNA) are short, single-stranded ~ 22-nucleotides-long RNA molecules, which are partially complementary to one or more messenger RNA (mRNA) molecules known as target mRNAs [1]. In humans, hundreds of miRNA genes are predicted to be present, and so, the potential regulatory circuitry afforded by miRNA is huge [2, 3]. They can either downregulate the gene expression [4] or can also upregulate the translation of mRNAs [5]. The expression of almost 20–30% of all protein-encoding genes may be altered by miRNAs at this post-transcriptional level regulation [6]. miRNAs may act as key regulators of processes as miscellaneous as embryonic development, cell proliferation, cell growth, tissue differentiation, and apoptosis. Recent studies of miRNA expression involve miRNAs in cellular signaling networks and co-regulation with transcription factors. Accordingly, a mutation in miRNAs, dysfunction of miRNA biogenesis, and dysregulation may result in a broad spectrum of diseases. In addition, components required for miRNA processing and/or function have also been implicated in various disorders. Currently, there have been reported ~ 378 diseases which are associated with miRNAs [7].

Intronic miRNAs can be defined based on two factors; first, they must share the same promoter with their encoded target genes, and second, they are spliced out of the transcript of such encoded genes and further processed into mature miRNAs [8]. About 37% of the known human miRNAs are located within the introns of protein-coding genes preferably known as host genes [9]. About 26% of the human intronic miRNAs are transcribed from their own promoters [10]. But the majority of human intronic miRNAs are transcriptionally linked to their host gene expression and processed from the same primary transcript [11]. In humans, most of the intronic miRNAs also show correlated expression with their host genes [12]. Besides Drosha-processed miRNAs, the second type of intronic miRNAs, mirtrons are discovered that bypass the Drosha cleavage by splicing [13] but exhibit the same co-expression patterns with their host genes.

Intronic miRNAs can negatively regulate their host genes by targeting the 3′-UTR of their host gene, inhibiting the host gene’s targets, or inhibiting the transcription of their host genes by a negative feedback loop. On the other hand, some intronic miRNAs can act as a positive regulator of their host gene by forming a positive feedback loop that upregulates the function of its host gene, working in concert with the host gene’s targets, or silencing antagonistic genes to its host gene [14].

Previous findings [15, 16] showed that the coupled expression of intronic miRNA and host gene were observed where miRNA can modulate the function of its host gene. Moreover, Steiman-Shimony et al. showed that intronic miRNAs may target transcripts whose genes/proteins are targeted by the host gene which can code a transcription factor [17], but no functional consequence of those interplays was revealed. Experiments to elucidate the functional regulations of the host gene and intronic miRNA were done in minuscule to date as they are time-consuming and extravagant [15, 16].

Like many other regulatory miRNAs, an important association of intronic miR-933 in various diseases like dementia [18], hyperlipidemia and cardiovascular diseases [19], and gastric cancers [20] was reported. It was also found that *ATF2* and miR-933 share a common promoter [21]. It is evident from the previous studies that the ATF2 transcription factor is involved in many diseases ranging from inflammatory diseases, diabetes, multiple neurodegenerative pathologies, different forms of cancers, etc. [22]. But the link between intronic miR-933 and host gene *ATF2* still remained elusive.

Although there are several intronic miRNAs that exist in the human genome, systemic identification of intronic miRNAs and their role in normal physiology and disease pathobiology is only explored in few cases [14]. In this study, after genome-wide identification of all probable intronic miRNAs, we explored the relationship between intronic miRNA hsa-miR-933 and its host genes with a combination of functional enrichment analysis to elucidate the functional relationship between them and the mechanism of these functional regulations. Furthermore, it was anticipated that the host gene’s and intronic miRNA’s common target gene sets for a particular biological process or pathway can act antagonistically or synergistically.

## Results

### A total of 822 intronic miRNAs and their associated host genes were identified

**Fig. 1:**
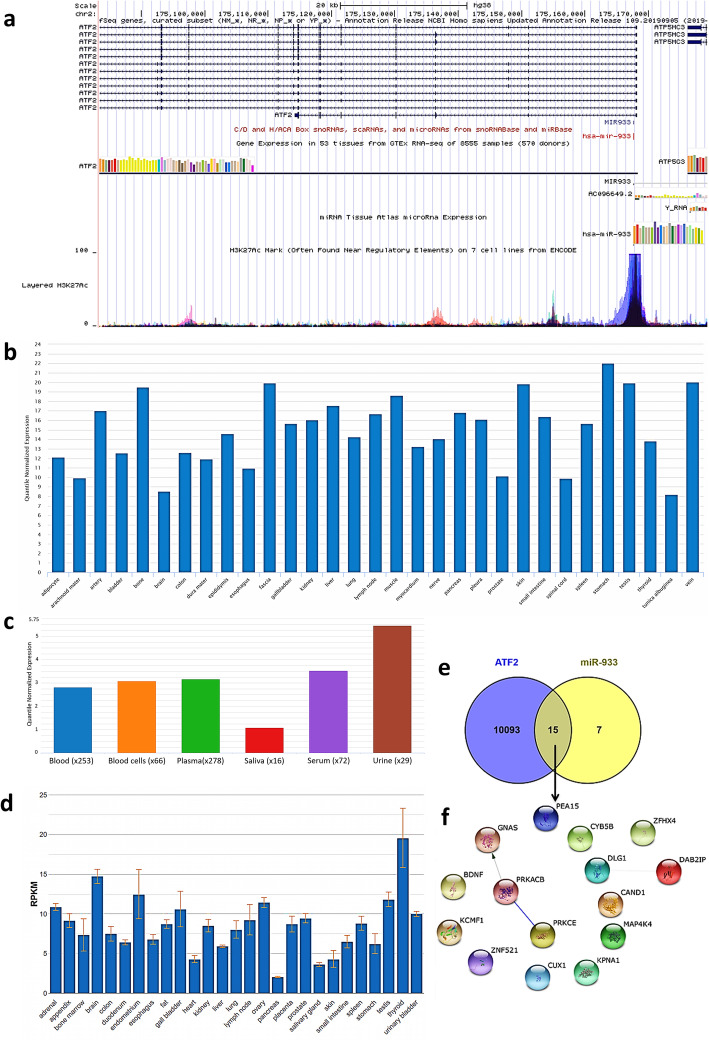
**a** UCSC Genome Browser view of *ATF2* and miR-933. miR-933 expression in **b** different tissue types and **c** different bodily fluids. **d** *ATF2* expression in different tissue type. **e** Common target genes of ATF2 and hsa-miR-933. **f** The PPI network shows an existing connection between PRKCE, PRKACB, and GNAS

From the human genome, we identified a total of 822 intronic miRNAs (data not shown). Among these, hsa-miR-933 was identified as embedded in the first intron of the host protein-coding gene activating transcription factor 2 (ATF2). ATF2 is a protein that acts as a transcriptional activator which regulates the transcription of a variety of genes. We also observed that host gene *ATF2* and miR-933 transcripts share common promoters (Fig. 1a) and are expressed in many similar tissue types (Fig. 1b–d). H3K27ac, an activating histone modification mark, is found to be enriched around the shared promoter of *ATF2* and miR-933 (Fig. 1a).

### A unique set of hsa-miR-933 targets identified from various target prediction algorithms

To enumerate the targets of hsa-miR-933, we have utilized the targets extracted from various microRNA target databases. From all these databases, we have identified the commonly predicted unique set of twenty-two genes to be targeted by the hsa-miR-933 (Additional file 2).

### Host (ATF2) target genes were determined from ChIP-seq experiments

ChIP-seq experiment in the cell line GM12878 from the ENCODE database [23] provides binding sites of ATF2 protein. For peak-to-target gene annotation, we utilized the well-known target-calling method “closestBed” implemented in BEDTools [24]. A similar algorithm is also utilized by another target-calling program ChIPpeakAnno [25]. We have identified candidate target genes of ATF2 by analyzing its ChIP-seq peaks and the complete dataset of *Homo sapiens* 149,604 transcripts of protein-coding genes as downloaded from Ensembl (release 70) [26]. By this approach, we have obtained a set of 10,108 targets of ATF2 (Additional file 3).

### Common targets of ATF2 and hsa-miR-933 reveal a connection between protein GNAS, PRKACB, and PRKCE

Next, to identify what are the genes that could be regulated by both host (ATF2) and intronic miRNA-933, we sought to determine the common target gene of both intronic miRNA-933 and its host gene *ATF2*, by Venn diagram overlap analysis. We have found that 15 common genes are targeted significantly by both hsa-miR-933 and ATF2 (*χ*^2^ = 37.9891, *p* value < 0.00001) (Additional file 5). These genes are *PEA15*, *ZFHX4*, *DAB2IP*, *CYB5B*, *DLG1*, *CAND1*, *MAP4K4*, *KPNA1*, *CUX1*, *ZNF521*, *KCMF1*, *BDNF*, *GNAS*, *PRKACB*, and *PRKCE* (Fig. 1e).

These 15 targets were subjected to protein-protein interaction (PPI) network analysis which revealed a connection between GNAS, PRKACB, and PRKCE proteins (Fig. 1f). This information was used to cluster GO annotations and KEGG pathway enrichment analysis.

### Intronic miRNA hsa-miR-933 regulates hyperinsulinemia and hyperglycemia in type II diabetes mellitus that may be caused by overexpression of ATF2

**Fig. 2:**
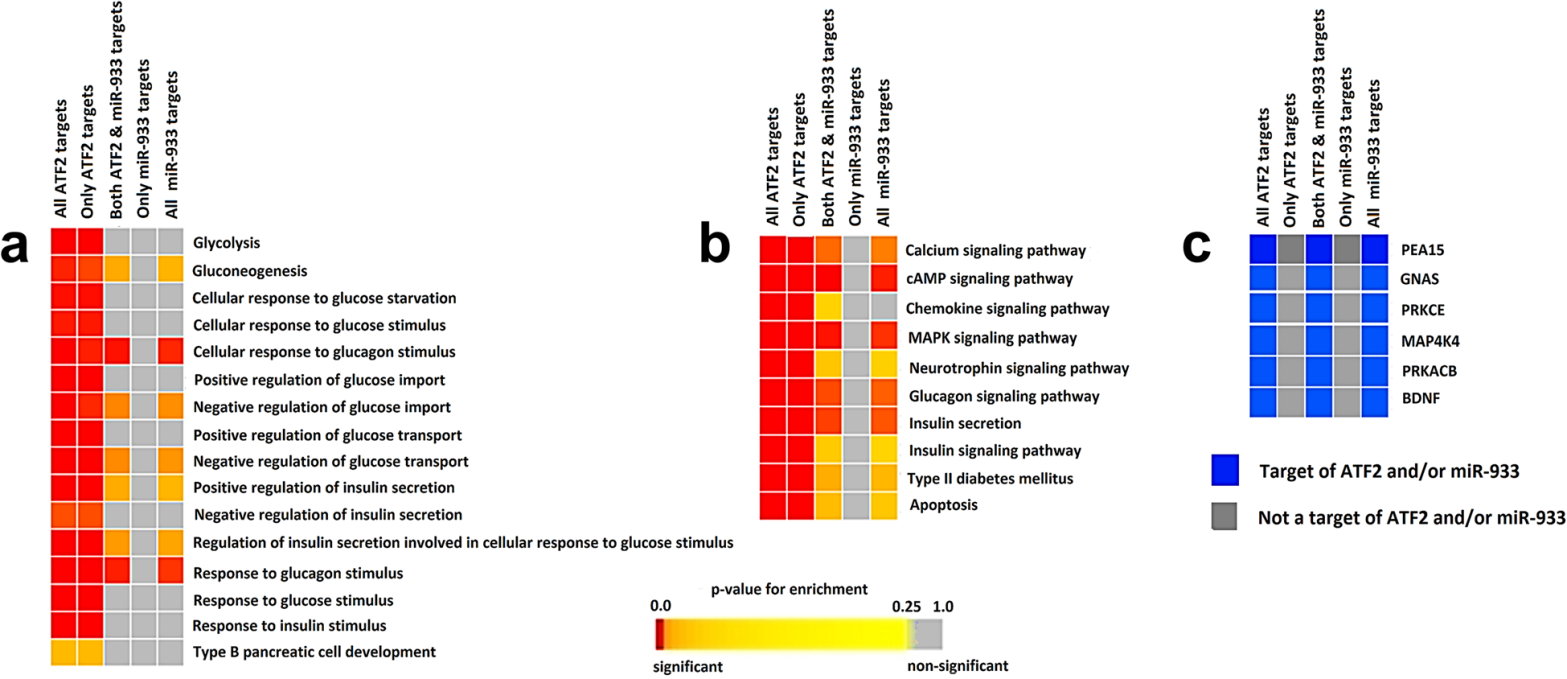
Enrichment of hsa-miR-933 and ATF2 target genes showing an over-representation of the terms related to type II diabetes mellitus. **a** Enrichment GOBP (GO biological process). **b** Enrichment of KEGG pathways. **c** Genes that are significantly over-represented in both categories for glucose homeostasis disruption. Selected significant terms are represented in the color-coded heatmap. The significance of enrichment in terms of the adjusted *p* value (< 0.05) is represented in the *p* value scale for all heatmaps. Color towards red indicates higher significance, and color towards yellow indicates less significance, while gray means non-significant

In order to illuminate the roles of the genes targeted by intronic miRNA hsa-miR-933, we have performed the enrichment analysis using the target genes for both ATF2 and hsa-miR-933. Significant enrichment (FDR ≤ 0.05) of the pathways necessary for the development of type II diabetes mellitus in terms of GO biological process (Fig. 2a) and KEGG pathways (Fig. 2b) was observed. Functional enrichment analysis showed significant enrichment of biological processes that can disrupt the normal glucose, insulin, and glucagon homeostasis in the cell by positively and negatively regulating them leading to the development of type II diabetes mellitus (Fig. 2a, b). The target genes that were significantly enriched for the development of type II diabetes mellitus in both modules are *GNAS*, *PRKACB*, *PRKCE*, *MAP4K4*, *PEA15*, and *BDNF* (Fig. 2c). The functions of the enriched genes are listed in Additional file 4.

**Fig. 3:**
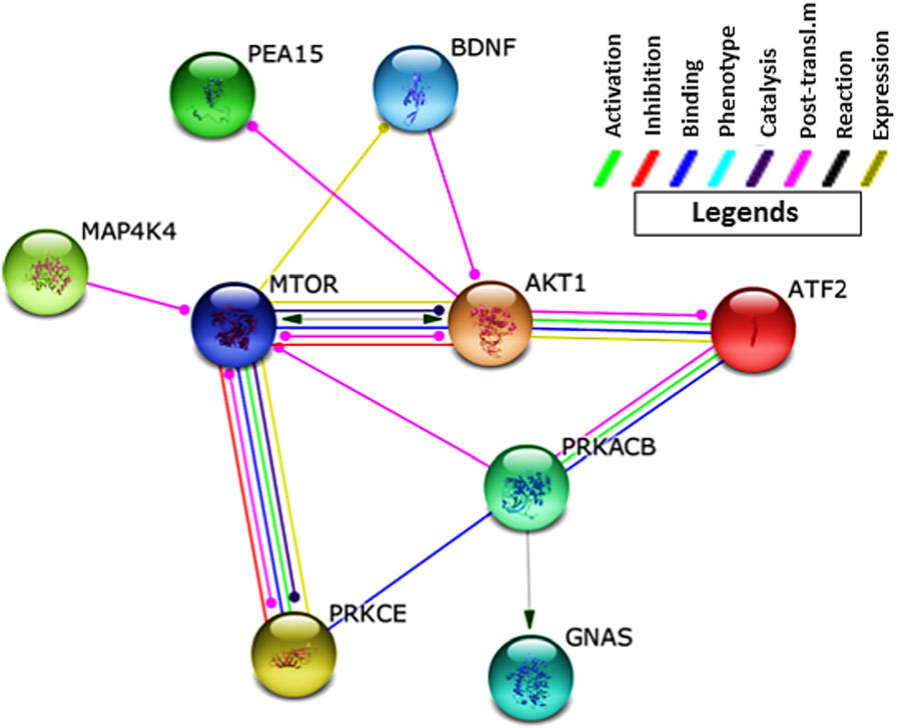
Putative regulatory network for the development of type II diabetes mellitus by target genes of ATF2 and hsa-miR-933. Modes of action are shown in different colors

**Fig. 4:**
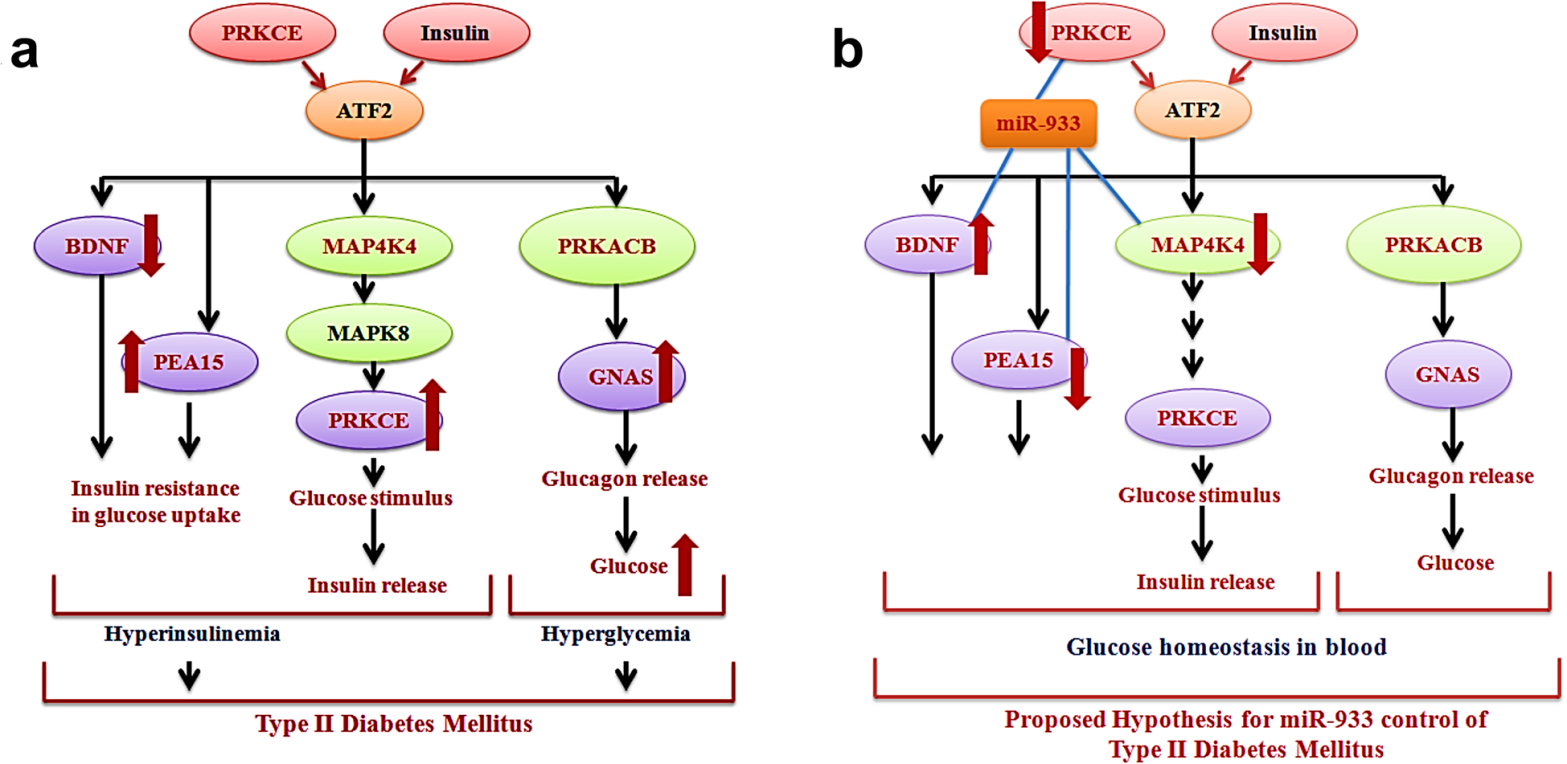
Schematic representation of the **a** projected mechanism for the development of type II diabetes mellitus mediated by hyperinsulinemia and hyperglycemia by ATF2 target genes *GNAS*, *MAP4K4*, *PRKACB*, *PRKCE*, *PEA15*, and *BDNF*. **b** Putative regulatory circuitry for controlling type II diabetes mellitus by regulating ATF2 target genes *MAP4K4*, *PRKCE*, *PEA15*, *BDNF*, *PRKACB*, and *GNAS* by hsa-miR-933

The network analysis using these common target genes shows ATF2-mediated activation of three genes *PRKACB*, *PRKCE*, and *ATK1*. PRKACB activates GNAS which promotes glucagon secretion upon glucose starvation. On the contrary, PRKCE and also MAP4K4 activate ATK1 (Fig. 3). ATK1 inhibits the expression of PEA15, an overexpressed protein in type II diabetes mellitus, where it may contribute to insulin resistance in glucose uptake. Hyperinsulinemia and hyperglycemia are both the pathophysiologies of type II diabetes mellitus (Fig. 4a). From this information, it can be considered for the analysis if hsa-miR-933 can regulate the overexpressed *PEA15*, downregulated *BDNF*, or other ATF2 target genes (*PRKACB*, *GNAS*, *PRKCE*, and *MAP4K4*) to control type II diabetes mellitus (Fig. 4b).

ATF2 transcription factor has been identified as an important component of the insulin signaling system and in maintaining glucose homeostasis in the cell [27]. ATF2 targets genes are involved in insulin action, β cell function, and type II diabetes mellitus. The other functions of ATF2 target genes include adipocyte dysfunction, inhibition of insulin signaling, lipid metabolism, glucose metabolism, β cell dysfunction, and many others [28–30]. ATF2 can also regulate gluconeogenesis for glucose production from the liver in response to glucose starvation [31]. Some ATF2 target genes, for example, BDNF and PEA15, are also linked to the development of insulin resistance under glucose stimulation [32, 33]. On the other hand, insulin itself activates ATF2 by phosphorylation of Thr69 and Thr71 [34] by the c-Jun/MAPK pathway. Improper activation of ATF2 target genes and ATF2 itself under conditions of insulin resistance can contribute to the development of type II diabetes mellitus [28].

In recent times, microRNAs are considered as a possible biomarker or a potential therapeutic for treating type II diabetes mellitus [35]. With this correspondence, the role of intronic miRNA hsa-miR-933’s functional regulation on its host gene ATF2 can provide valuable insight into the progression and control of type II diabetes mellitus.

### hsa-miR-933 plays a role in neuronal regeneration and protection by suppressing the overexpressed targets of host gene *ATF2* responsible for neuron death

**Fig. 5:**
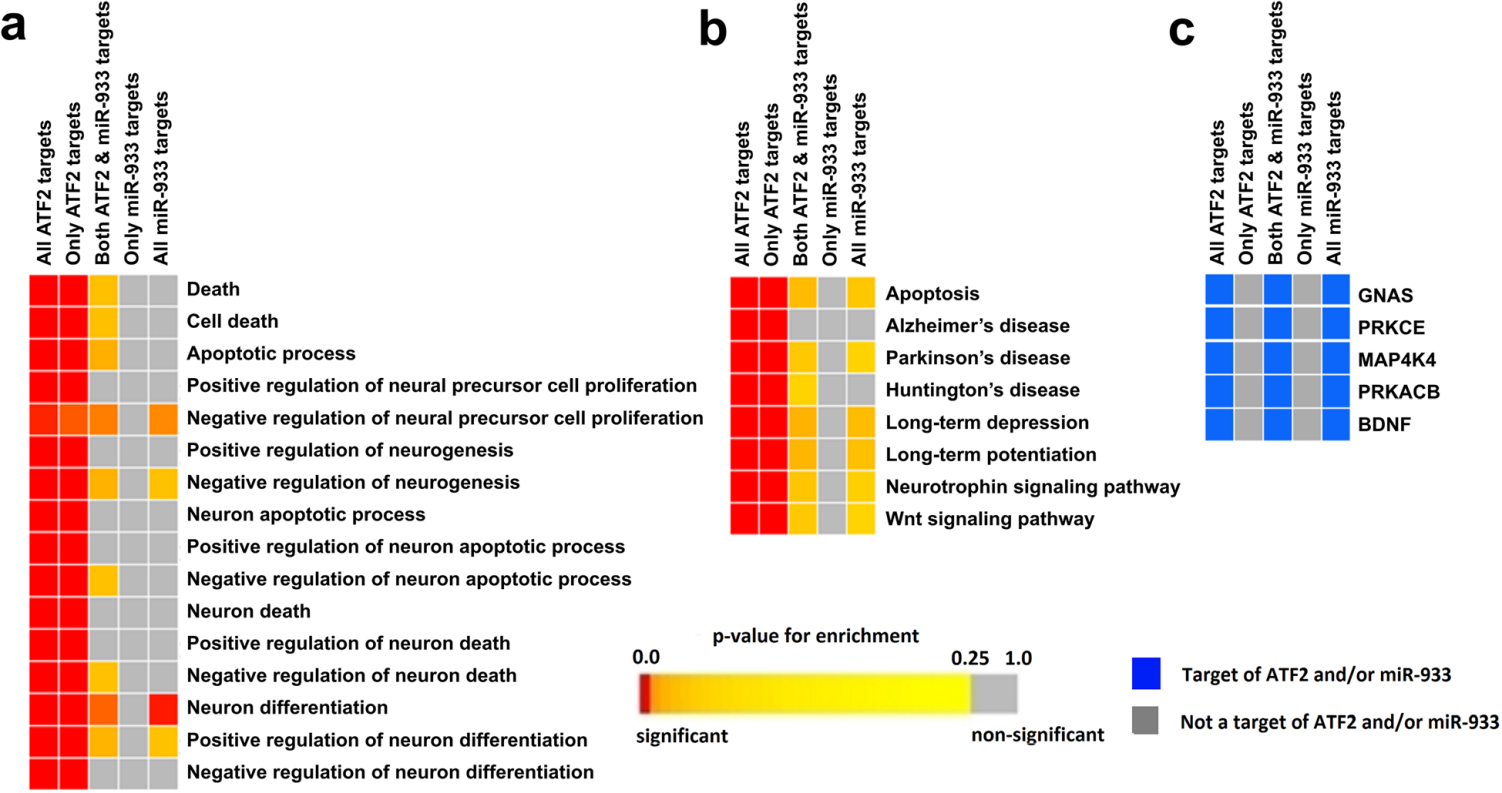
Enrichment of hsa-miR-933 and ATF2 targets showing an over-representation of neuronal degeneration. **a** Enrichment GOBP (GO biological process). **b** Enrichment of KEGG pathways. **c** Genes that are significantly over-represented in both categories. Selected significant terms are represented in the color-coded heatmap. The significance of enrichment in terms of the adjusted *p* value (< 0.05) is represented in the *p* value scale for all heatmaps. Color towards red indicates higher significance, and color towards yellow indicates less significance, while gray means non-significant

In order to reveal whether the targets of ATF2 and hsa-miR-933 are involved in other significant pathways, we analyzed the functional enrichment using the GOBP module of the target genes. Interestingly, we have observed a significant enrichment of biological processes that contrast between the positive and negative regulatory cycles of neuron apoptotic process (Fig. 5a). Enrichment analysis with the KEGG pathway module also shows the significant pathways necessary for the development of different diseases related to neuron degradation, for example, Huntington’s disease, Parkinson’s disease, and long-term depression (Fig. 5b). The target genes that were significantly enriched in both modules are *GNAS*, *PRKACB*, *PRKCE*, *MAP4K4*, *PEA15*, and *BDNF* (Fig. 5c). The functions of the enriched genes are listed in Additional file 4.

**Fig. 6:**
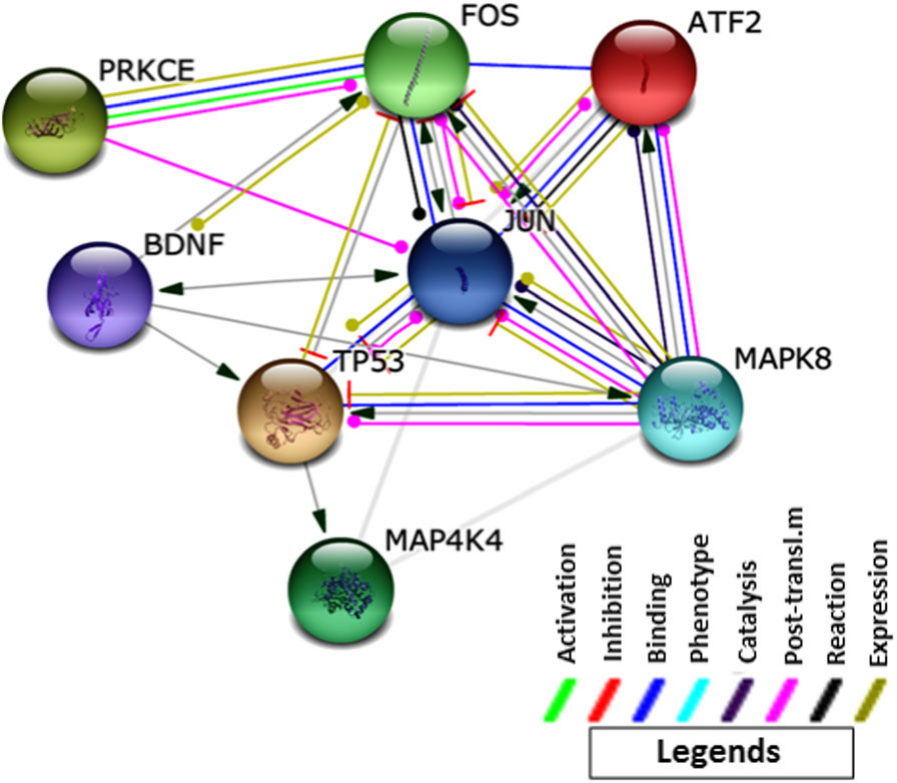
Putative regulatory network for neurodegenerative disease by ATF2 and miR-933 target genes. Modes of action are shown in different colors

**Fig. 7:**
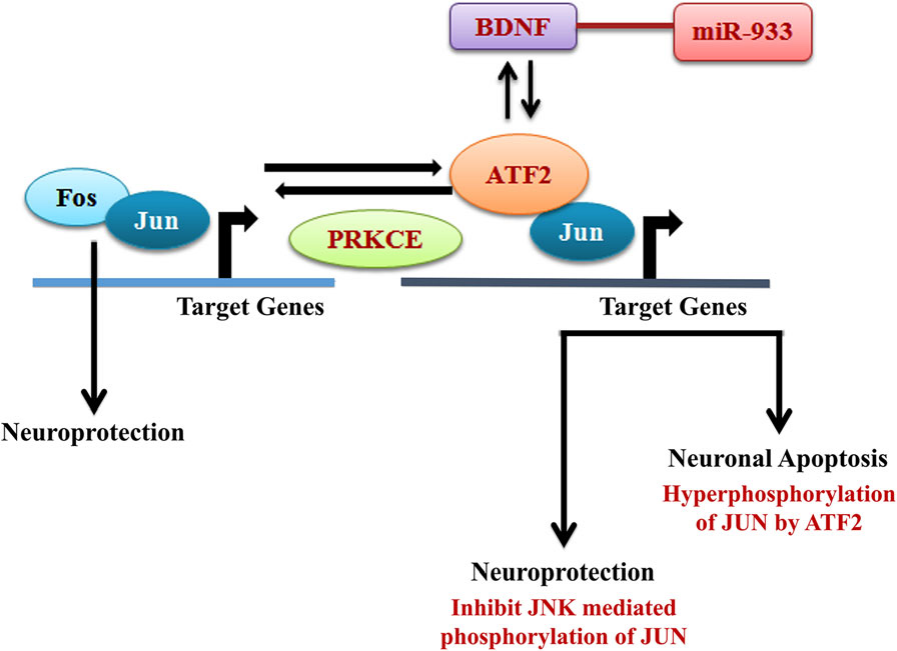
Putative controlling model of neurodegeneration by regulating ATF2 target genes *PRKCE* and *BDNF* by miR-933

From network analysis, we have tracked down the involvement of ATF2 in the activation of two genes: *BDNF* via JUN; *PRKCE* via FOS. PRKCE directly inhibits JUN thus repressing the expression of *BDNF* leading to neurodegeneration (Fig. 6). On the other hand, recently, it was discovered that hsa-miR-933 is a target of *BDNF* that may be involved in cell growth, apoptosis, cell proliferation, or the regulation of the cell cycle. It is thus to be analyzed if hsa-miR-933 can act as a potential repressor of neurodegeneration or promote neurodegeneration (Fig. 7).

*ATF2* is highly expressed with large variations in the brain (Fig. 1d) and plays a role in both neurodegeneration and neurogenesis. ATF2 seems to play a fundamental role in neuronal viability and in neurological functions in the normal brain. ATF2 is downregulated in the hippocampus and the caudate nucleus in Alzheimer’s, Parkinson’s, and Huntington’s diseases [36].

ATF2/JUN heterodimers bind and activate CASP3, a key executor of neuronal apoptosis [37]. Following death receptor stimulation, there is phosphorylation and binding of ATF2/JUN to death-inducing ligand promoters (FASLG, TNF, TNFSF10), which allows the spread of death signals [38]. Neuronal apoptosis requires the simultaneous activation of ATF2/JUN and downregulation of FOS [39]. This function is negatively regulated by phosphorylation of ATF2 by PRKCE, which dictates its nuclear localization [40].

Loss of functional ATF2 leads to hyperphosphorylated JNK and p38 which results in somatic and visceral motor neuron degeneration [41]. On the other hand, activated ATF2 promotes apoptosis of various brain cells, which are cerebellar granule neurons [37]. ATF2/JUN heterodimers also promote the death of sympathetic neurons [39]. Sometimes, ATF2/JUN inhibits JNK-mediated phosphorylation of JUN and protects sympathetic neurons from apoptosis [42].

Another ATF2 target BDNF can play a neuroprotective role against neural apoptosis [43]. Moreover, recently, brain-derived neurotrophic factor (BDNF) has been identified as a possible target sequence for hsa-miR-933 [44]. So, exploiting this information, it can be analyzed if ATF2 and hsa-miR-933 can play a neuroprotective role in neurodegenerative diseases by regulating their common target gene *BDNF*.

## Discussion

In recent times, biological modifier microRNA (miRNA) has captured extensive attention for being a potential candidate for studying their role in the regulation of gene expression to delineate a variety of physiological processes related to the progression and pathogenesis of human diseases.

Almost half of the experimentally identified human miRNAs are encoded in the introns of annotated protein-coding genes which can be preferably denoted as intronic miRNA embedded within the host gene. Along with the breakthrough discovery of important functions for these intronic miRNAs, the fact that made research in this field of such significance is intronic miRNAs are co-expressed and share similar transcriptional regulatory mechanisms with their host genes in humans. This parallel expression pattern suggests that these intronic miRNAs may have functions either similar or opposite to that of their host genes in a cell type- or tissue type-specific spatial manner or expression time-specific temporal manner.

Despite the mounting evidence for their importance in normal physiology, very little is known about the regulatory cascade controlled by intronic miRNA and host gene in humans to date. In this study, a genome-wide network-based and data mining approach was adopted to elucidate the functional relationship between intronic miRNAs and host gene, and the effects of intronic miRNAs on host gene targets to control cellular homeostasis.

ATF2 is activated by stress kinases, including JNK and p38. In response to stimuli, ATF2 is phosphorylated on threonine 69 and/or 71 by JNK or by p38. Phosphorylation on Thr69 and Thr71 of ATF2 and its dimerization are required to activate ATF2 transcription factor activity [30]. At the same time, transcriptionally active dimers of ATF2 protein are regulated by ubiquitylation and proteasomal degradation [45], and phosphorylation of ATF2 on Thr69 and Thr71 promotes its ubiquitylation and degradation [46]. This phosphorylation and dephosphorylation cascade is an important factor for the positive regulation of human insulin gene expression [47].

Hyperinsulinemia is a condition when the amount of insulin circulating in the blood is higher than expected. Insulin itself can create a positive feedback loop by activating ATF2 in this pathway solely or mediated by MAPK8 (preceded by MAP4K4) or PRKCE which can eventually lead to hyperinsulinemia [34]. Insulin overproduction also leads to the overexpression of other ATF2 target genes that lead to β-pancreatic cell dysfunction—pathophysiology of type II diabetes mellitus [48].

One of the ATF2 target gene—*MAP4K4* is an attenuator for insulin signaling [49]. MAPK4K4 also plays an important role in insulin resistance in response to glucose stimulus for the development of type II diabetes mellitus [50]. *GNAS* and *PRKACB* are another two ATF2 target genes that are involved in the regulation of glucagon secretion [51, 52] and insulin secretion for maintaining glucose homeostasis in cells. Impairment in this regulation can lead to hyperglycemia, and the resultant excess glucose in the bloodstream leads to the development of type II diabetes mellitus [52]. Other ATF2 target genes *BDNF* downregulation and *PEA15* upregulation are also linked to the development of insulin resistance under glucose stimulation, pathophysiology of type II diabetes mellitus [32, 33].

All these genes show significant enrichment in the functional enrichment analysis performed in our study with ATF2 and hsa-miR-933. Thus, hsa-miR-933 can be considered as a potential regulator of type II diabetes mellitus. Recent researches are more focused on considering microRNAs as the new therapeutics to treat type II diabetes mellitus [53]. Recently, it has also been discovered that microRNA-30d can induce insulin production by targeting *MAP4K4* [54]. microRNAs 103/107 can regulate insulin sensitivity [55]. In this context, it can be considered if hsa-miR-933 can also regulate *MAP4K4* or other protein kinases (*PRKCE*, *PRKACB*) to control type II diabetes mellitus [56]. Also, recently, brain-derived neurotrophic factor (*BDNF*) has been identified as a possible target sequence for hsa-miR-933 [44]. So, it can also be considered if hsa-miR-933 can reverse the effect of insulin resistance in response to glucose stimulus by upregulating *BDNF*.

The significant over-representation of genes associated with type II diabetes mellitus, related pathways, and associated biological processes identified from our study, indicates the firm possibility of considering hsa-miR-933 as a potential regulator of treating the complications linked with the disease.

ATF2 transcription factor is a ubiquitously expressed protein in humans with a more abundant expression in the brain. This profuse expression enables it to have a key role in both neurodegeneration and neurogenesis [36]. This transcription factor also has a function in neuronal migration during development [57]. But overexpression of *ATF2* in neuronal-like cell culture promotes nerve cell death [36]. In addition, ATF2 has an essential role in neuronal viability and in neurological functions in the normal brain. In neurodegenerative diseases like Alzheimer’s, Parkinson’s, and Huntington’s diseases, ATF2 is downregulated in the hippocampus and the caudate nucleus [36]. So, the proper regulation of ATF2 is necessary for the neurons in the brain’s central nervous system.

Activating transcription factor 2 (ATF2) is a member of the activator protein-1 family of transcription factors that promote neuronal apoptosis by c-Jun and c-Fos cascade in the cytoplasm. Neuronal apoptosis requires the simultaneous activation of ATF2/c-JUN and downregulation of c-FOS [39]. This function is negatively regulated by phosphorylation of ATF2 by PRKCE, which dictates its nuclear localization [40]. Loss of functional ATF2 leads to hyperphosphorylated JNK and p38 and results in somatic and visceral motor neuron degeneration [41]. Sometimes, activated ATF2 also promotes apoptosis of various brain cells, for example, cerebellar granule neurons [37].

ATF2/c-JUN heterodimers also promote the death of sympathetic neurons [39, 42]. In a negative feedback loop, ATF2/c-JUN also inhibits JNK-mediated phosphorylation of JUN and protects sympathetic neurons from apoptosis [42]. Additionally, ATF2 can also play a role in neurogenesis by maintaining a subset of neural progenitor cells [58]. Another ATF2 target *BDNF* has a neuroprotective role against neural apoptosis [43, 59].

Considering this fact, ATF2 can be presented as an important regulator of nervous system viability. The most recent research field, treating neurodegenerative diseases with microRNAs, can be used to modulate any aberrant expression of ATF2 target genes and subsequent abnormality leading to neuronal degradation or to provide sufficient neuronal protection [60–62]. As stated earlier, *BDNF* which is also over-represented in the enrichment analysis of this study of neurodegenerative disease has been identified as a possible target sequence for hsa-miR-933 [44]. So, exploiting this information, it can be analyzed whether ATF2 and hsa-miR-933 can play a neuroprotective role in neurodegenerative diseases by regulating their common target gene *BDNF*.

Our study of functional enrichment analysis sheds light on the functional interactions between intronic miRNAs and host genes. Therefore, these findings can have potential applications in the development of diagnostic and treatment methods.

## Conclusion

The outcome of this study shows that intronic miRNAs and their host genes can coincide with functional relations. Using a data-driven as well as a knowledge-based computational approach, common targets of intronic miRNA and their associated host genes were analyzed for functional co-relations. A further GO analysis predicted an intronic miRNA-host gene interaction network that confirms that the predicted target genes tend to be regulated simultaneously. Taken together, these results indicate either synergistic or antagonistic regulatory effects mediated by either downregulation of genes with an opposed function or fine-tuning of intronic miRNA targets, co-operative to the host gene.

## Methods

### Identification of host gene of hsa-miR-933

Genomic location position data of *Homo sapiens* microRNA hsa-miR-933 and protein-coding genes were retrieved from Ensembl (release 70) [26] database using the BioMart portal. We used the IntersectBed feature of the BEDTools [24] to find the host gene in which the hsa-miR-933 is embedded. We also cross-checked the promoter of the transcripts of hsa-miR-933 using the web portal http://fantom.gsc.riken.jp/5/suppl/De_Rie_et_al_2017/ [21].

### Obtaining the tissue-wide expression profiles of hsa-miR-933 and *ATF2*

We have retrieved the tissue-wide expression data of the host gene *ATF2* from NCBI Bioproject ID: PRJEB4337. Human miRNA tissue atlas [63] was used to obtain the gene expression data of hsa-miR-933 in different tissue types and bodily fluids.

### Identification of the targets of the host gene (*ATF2*)

After identifying the host gene, we have identified the targets of the host gene. For this, ChIP-seq experiment datasets (Additional file 1) of the host gene were retrieved from the ENCODE database [64] for analysis. For target calling, we used the “closestBed” feature of the BEDTools [24] to identify the nearest transcript from the ChIP-seq peak as the target gene using Ensembl (release 70) transcripts.

### Identification of the targets of hsa-miR-933

We have extracted the targets of hsa-miR-933 from several miRNA target gene databases which include both experimental and predicted targets, namely TargetScan v6.2 [6], TarBase v4 [65], PITA v6 [66], PicTar [67], experimentally validated miRTarBase v4.5 [68], miRBase [69], and miRecords v4 [70]. A commonly predicted miRNA target gene set was prepared from the targets retrieved from different microRNA target databases for further analysis.

### Identification of common targets of intronic miRNA and host gene by overlap analysis

To find out the common targets of hsa-miR-933 and its host gene, we have conducted an overlap analysis using the interactive tool Venny (v 2.0.2) [71]. The result generated the list of targets that are both targeted by the host gene and hsa-miR-933.

### Functional enrichment analysis

The functional annotation of target genes is based on Gene Ontology (GO) [72] as extracted from the Ensembl database (release 70) [26] and KEGG pathway database [73]. Accordingly, all genes are classified into the ontology categories biological process (GOBP), cellular component (GOCC), molecular function (GOMF), and pathways when possible. The GO/pathway categories that have at least 10 genes annotated were only considered. Gitools [74] was used for functional over-representation analysis and heatmap generation. Resulting *p* values were adjusted for multiple testing using the Benjamin and Hochberg’s method of false discovery rate (FDR) [75].

### Clustering of biological processes and pathways with a functional similarity of host genes and intronic miRNA target gene sets

The host gene confers regulatory control by transcriptional or translational inhibition/activation of the hsa-miR-933 target genes or vice versa in possibly related biological processes or pathways. To test this hypothesis for all hosts and target genes, their respective annotations were compared and clustered manually based on the literature. Functional gene annotations as provided by the GO [72] and KEGG pathway [73] classify genes according to their molecular function, associated biological processes, or appearance within defined cellular components and pathways they appear.

### Construction of network with enriched gene sets derived from functional enrichment analysis

We have constructed the protein-protein interaction networks of enriched targets of the host gene and the miRNA hsa-miR-933 using tools STRING (version 10) [76] for possibly related biological processes or pathways. Based on protein-protein interaction, and data and knowledge-driven approach, the hypothesis for a particular enriched biological process or pathway was predicted.

The whole workflow of this study is summarized in Additional file 6: Figure S1.

## Supplementary information

**Additional file 1.** ChIP-seq Experiment data downloaded from ENCODE.

**Additional file 2.** List of genes targeted by hsa-miR-933.

**Additional file 3.** Partial list of 160 targets from a total of 10108 targets of the host gene ATF2.

**Additional file 4.** Functions of the enriched genes for the development of hyperinsulinemia; and Neurodegeneration.

**Additional file 5: **Contingency table of the Chi-square test for finding the statistical significance of the overlapped targets of miR-933 and *ATF2*.

**Additional file 6: Figure S1.** Whole workflow of the study.

## Data Availability

Datasets used in the analyses can be found in various publicly available databases.

## Abbreviations

miRNA: MicroRNA
TF: Transcription factor
TSS: Transcription start site
GO: Gene Ontology
GOBP: GO biological process
KEGG: Kyoto Encyclopedia of Genes and Genomes
FDR: False discovery rate
