# Supplementary material for: Aberration of the modulatory functions of intronic microRNA hsa-miR-933 on its host gene ATF2 results in type II diabetes mellitus and neurodegenerative disease development
Source: Hum Genomics. 2020 Sep 29;14:34. doi: 10.1186/s40246-020-00285-1 (PMC7526404; doi:10.1186/s40246-020-00285-1)
Supplement: Supplementary file 1 — Additional file 1. ChIP-seq Experiment data downloaded from ENCODE. [file 40246_2020_285_MOESM1_ESM.docx]

**Additional file 1: ChIP-seq Experiment data downloaded from ENCODE.**

| **Host gene** | **Cell Line (Tissue/Karyotype)** | **Encode ChIPseq data source Lab** |
| --- | --- | --- |
| **ATF2** | GM12878 (blood, normal) | Myers - Hudson Alpha |
|  | H1-hESC (embryonic stem cell, normal) | Myers - Hudson Alpha |
