# Supplementary material for: Aberration of the modulatory functions of intronic microRNA hsa-miR-933 on its host gene ATF2 results in type II diabetes mellitus and neurodegenerative disease development
Source: Hum Genomics. 2020 Sep 29;14:34. doi: 10.1186/s40246-020-00285-1 (PMC7526404; doi:10.1186/s40246-020-00285-1)
Supplement: Supplementary file 2 — Additional file 2. List of genes targeted by hsa-miR-933. [file 40246_2020_285_MOESM2_ESM.docx]

**Additional file 2: List of genes targeted by has-miR-933.**

| **miRNA** | **Targeted genes** |
| --- | --- |
| **hsa-miR-933** | *BDNF*  *CAND1*  *COL12A1*  *CUTL1*  *CYB5B*  *DAB2IP*  *DAZAP1*  *DLG1*  *GNAS*  *HOXA1*  *KCMF1*  *KPNA1*  *MAP4K4*  *MGC20255*  *PEA15*  *POLR3H*  *PRKACB*  *PRKCE*  *SLC16A2*  *VPS4B*  *ZFHX4*  *ZNF521* |
