# Supplementary material for: Aberration of the modulatory functions of intronic microRNA hsa-miR-933 on its host gene ATF2 results in type II diabetes mellitus and neurodegenerative disease development
Source: Hum Genomics. 2020 Sep 29;14:34. doi: 10.1186/s40246-020-00285-1 (PMC7526404; doi:10.1186/s40246-020-00285-1)
Supplement: Supplementary file 3 — Additional file 3. Partial list of 160 targets from a total of 10108 targets of the host gene ATF2. [file 40246_2020_285_MOESM3_ESM.docx]

**Additional file 3: Partial list of 160 targets from a total of 10108 targets of the host gene ATF2.**

| AAAS | ABCB9 | ABT1 | ACPP | ACTRT3 | ADAMTS16 | ADCY2 | ADRM1 |
| --- | --- | --- | --- | --- | --- | --- | --- |
| AACS | ABCC1 | ABTB2 | ACPT | ACVR1 | ADAMTS17 | ADCY3 | ADSL |
| AADAT | ABCC11 | ACACA | ACSBG1 | ACVR1B | ADAMTS18 | ADCY7 | ADSS |
| AAGAB | ABCC12 | ACAD8 | ACSF3 | ACVR2A | ADAMTS20 | ADCY9 | ADTRP |
| AAK1 | ABCC4 | ACAD9 | ACSL1 | ACY3 | ADAMTS4 | ADD1 | AE000660.1 |
| AASDHPP | ABCC5 | ACADSB | ACSL3 | ACYP2 | ADAMTS6 | ADD3 | AE000662.92 |
| AATF | ABCD2 | ACBD4 | ACSL4 | ADA | ADAMTS7 | ADH5 | AEBP2 |
| AATK | ABCF2 | ACBD5 | ACSL5 | ADAM17 | ADAMTSL1 | ADH7 | AF070718.1 |
| AB019440.1 | ABCG1 | ACD | ACSL6 | ADAM19 | ADAMTSL3 | ADK | AF165138.7 |
| ABCA1 | ABHD11 | ACER2 | ACSM5 | ADAM2 | ADAP2 | ADNP | AF250324.1 |
| ABCA12 | ABHD12 | ACN9 | ACSS1 | ADAM21 | ADAR | ADNP2 | AFAP1 |
| ABCA13 | ABHD16A | ACO1 | ACSS2 | ADAM22 | ADARB1 | ADO | AFAP1L2 |
| ABCA3 | ABHD2 | ACO2 | ACTA2 | ADAM23 | ADARB2 | ADORA2B | AFF1 |
| ABCA4 | ABHD5 | ACOT11 | ACTB | ADAM28 | ADAT1 | ADORA3 | AFF2 |
| ABCA5 | ABI1 | ACOT7 | ACTG1 | ADAM32 | ADC | ADPRH | AFF3 |
| ABCA6 | ABI3 | ACOT9 | ACTN4 | ADAM8 | ADCK1 | ADPRHL2 | AFM |
| ABCB1 | ABLIM1 | ACOX3 | ACTR1B | ADAMDEC1 | ADCK2 | ADRB1 | AFTPH |
| ABCB11 | ABR | ACOXL | ACTR2 | ADAMTS12 | ADCK3 | ADRB2 | AGA |
| ABCB4 | ABRA | ACP1 | ACTR3 | ADAMTS13 | ADCY1 | ADRBK1 | AGAP1 |
| ABCB5 | ABRACL | ACP2 | ACTR6 | ADAMTS14 | ADCY10 | ADRBK2 | AGBL3 |
