# Supplementary material for: Aberration of the modulatory functions of intronic microRNA hsa-miR-933 on its host gene ATF2 results in type II diabetes mellitus and neurodegenerative disease development
Source: Hum Genomics. 2020 Sep 29;14:34. doi: 10.1186/s40246-020-00285-1 (PMC7526404; doi:10.1186/s40246-020-00285-1)
Supplement: Supplementary file 4 — Additional file 4. Functions of the enriched genes for the development of hyperinsulinemia; and Neurodegeneration. [file 40246_2020_285_MOESM4_ESM.docx]

**Additional file 4: Functions of the enriched genes for the development of hyperinsulinemia; and Neurodegeneration.**

| **Gene Name** | **Function** |
| --- | --- |
| *GNAS* | GNAS complex locus; Guanine nucleotide-binding proteins (G proteins) are involved as modulators or transducers in various transmembrane signaling systems. The G(s) protein is involved in hormonal regulation of adenylate cyclase- it activates the cyclase in response to beta-adrenergic stimuli. |
| *PRKACB* | Mediates cAMP-dependent signaling triggered by receptor binding to GPCRs. PKA activation regulates diverse cellular processes such as cell proliferation, the cell cycle, differentiation and regulation of microtubule dynamics, chromatin condensation and decondensation, nuclear envelope disassembly and reassembly, as well as regulation of intracellular transport mechanisms and ion flux. Regulates the abundance of compartmentalized pools of its regulatory subunits through phosphorylation of PJA2 which binds and ubiquitinates these subunits, leading to their subsequent proteolysis |
| *PRKCE* | Calcium-independent, phospholipid- and diacylglycerol (DAG)-dependent serine/threonine-protein kinase that plays essential roles in the regulation of multiple cellular processes linked to cytoskeletal proteins, such as cell adhesion, motility, migration and cell cycle, functions in neuron growth and ion channel regulation, and is involved in immune response, cancer cell invasion and regulation of apoptosis. |
| *MAP4K4* | Mitogen-activated protein kinase kinase kinase kinase 4; Serine/threonine kinase that may play a role in the response to environmental stress and cytokines such as TNF-alpha. Appears to act upstream of the JUN N-terminal pathway. Phosphorylates SMAD1 on Thr-322 |
| *BDNF* | During development, promotes the survival and differentiation of selected neuronal populations of the peripheral and central nervous systems. Participates in axonal growth, pathfinding and in the modulation of dendritic growth and morphology. A major regulator of synaptic transmission and plasticity at adult synapses in many regions of the CNS. The versatility of BDNF is emphasized by its contribution to a range of adaptive neuronal responses including long-term potentiation (LTP), long-term depression (LTD), certain forms of short-term synaptic plasticity, as well as homeostatic regulation of intrinsic neuronal excitability. |
| *PEA15* | Blocks Ras-mediated inhibition of integrin activation and modulates the ERK MAP kinase cascade. Inhibits RPS6KA3 activities by retaining it in the cytoplasm (By similarity). Inhibits both TNFRSF6- and TNFRSF1A-mediated CASP8 activity and apoptosis. Regulates glucose transport by controlling both the content of SLC2A1 glucose transporters on the plasma membrane and the insulin-dependent trafficking of SLC2A4 from the cell interior to the surface. |
