# Supplementary figures and images for: Aberration of the modulatory functions of intronic microRNA hsa-miR-933 on its host gene ATF2 results in type II diabetes mellitus and neurodegenerative disease development
Source: Hum Genomics. 2020 Sep 29;14:34. doi: 10.1186/s40246-020-00285-1 (PMC7526404; doi:10.1186/s40246-020-00285-1)

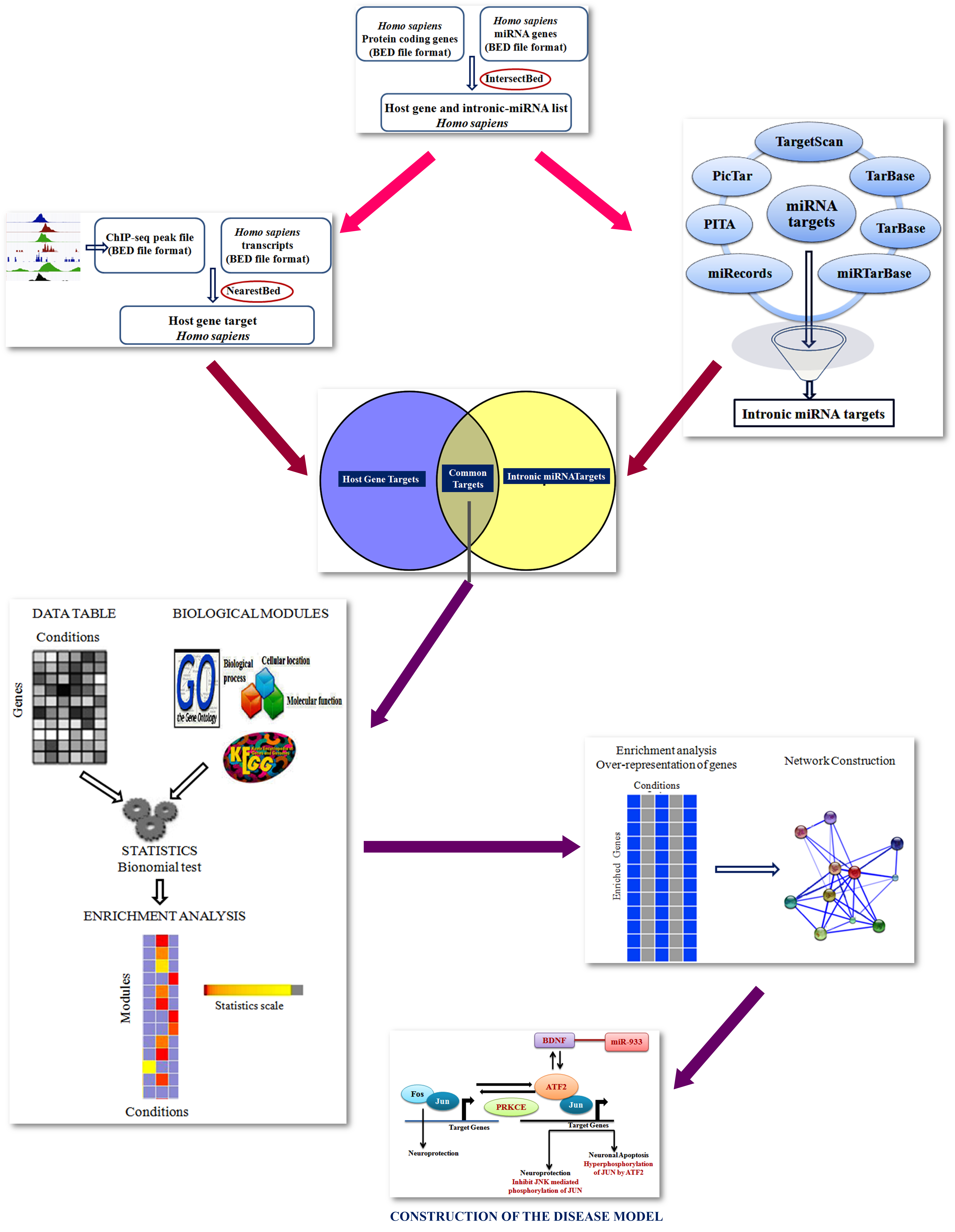

Supplement: Supplementary file 6 — Additional file 6: Figure S1. Whole workflow of the study. [file 40246_2020_285_MOESM6_ESM.tif]
